# Evolution of genotoxicity test methods in Japan

**DOI:** 10.1186/s41021-016-0063-7

**Published:** 2017-02-21

**Authors:** Toshio Sofuni

**Affiliations:** Formerly National Institute of Health Sciences, Tokyo, Japan

**Keywords:** Genotoxicity test methods, Food additives, AF-2

## Abstract

The evolution of methods to assess genotoxicity of test compounds is thought to be one of the important subjects in The Japanese Environmental and Mutagen Society (JEMS). In 1970, the Ministry of Education of Japan (at that time) organized a research group (Organizer: Y. Tazima, National Institute of Genetics), and started a systematic research on the genotoxic effects induced by chemical substances. Considering the importance of this issue through the outcomes of the research group, JEMS was established in 1972, and President Tazima organized the 1st annual meeting in the August in Tokyo with the participation of experts in this field working in national institutes, universities and others in Japan. The discovery that food additives possessed genotoxic potential triggered various scientific activities in the field of genotoxicity. Another important point was the correlation between genotoxicity and carcinogenicity, in which the establishment of the reverse mutation assay played an important role. Other critical factors, such as side effects of drugs, occupational cancer, and environmental pollution due to genotoxic chemicals, emphasized the importance of genotoxicity tests for human safety. The tests performed to assess genotoxicity from 1960s to 1980s will be described to understand that many different genotoxic methodologies were discussed in these periods.

## Introduction

Food additives and genotoxicity tests are interrelated, in which the most important food additive is 2-(2-furyl)-3-(5-nitro-2-furyl) acrylamide (AF-2). Initially, I would like to introduce the words written by M. Ishidate, Jr. who was the first Director of the Division of Genetics and Mutagenesis, National Institute of Health Sciences, Tokyo, Japan: “The safety of AF-2 used in tofu (bean curd) as a germicide was questioned in Japan a few years ago. Initially, geneticists pointed out that the AF-2 was mutagenic in microorganisms, such as bacteria, and was clastogenic in cultured human lymphocytes. Subsequently, its carcinogenicity in mice was demonstrated, after which administrative measures were taken to regulate the use of AF-2 [1].” In 1974, AF-2 was officially prohibited for use. Ishidate wrote “a few years ago,” although his document was published seven years after the prohibition, suggesting how great the impact of the AF-2 issue was.

At the beginning of 1970s, Prof. B. N. Ames developed, as a new test system for genotoxicity, the reverse mutation assay using *Salmonella typhimurium*, the so-called “Ames test.” His research group examined approximately 300 substances using Ames test and found a high correlation between carcinogenicity and genotoxicity, indicating that this test could be used for the screening of carcinogenic chemicals as it consumed less time. However, AF-2 initially showed negative results in Ames test with bacterial strains such as TA1535 and TA1538. Therefore, new and more sensitive test strains were constructed, such as TA100 and TA98, by introducing the plasmid pKM101 into TA1535 and TA1538 strains, respectively. Both TA100 and TA98 strains showed positive results with AF-2, particularly in TA100 [2]. The reason of the high sensitivity of TA100 is *mucAB* genes on pKM101 although they were not well studied at that time. About thirty years later, it was revealed that *mucAB* genes encode a DNA polymerase R1 related to translesion DNA synthesis, which causes mutation due to mis-incorporation of bases opposite DNA lesions generated by chemicals, such as AF-2. Consequently, five to six tester strains, including TA100 and TA98, were used as standards in routine testing for the reverse mutation assay. The relationship between genotoxicity tests and food additives is close, as mentioned above. I would like to describe an outline of the evolution of genotoxicity tests at the initial stage (1960’s to 1980’s).

## Outline of genotoxicity tests in the initial stage

Around 1945, certain chemicals were recognized to possess genotoxic potential, but the situation at such a preliminary stage is omitted here. In 1965, Food Sanitation Council in Japan published Standards for Designation of Food Additives and for Revision of Use Regulation of Food Additives, in which only two tests, namely, acute toxicity test and chronic toxicity test were adopted for toxicity evaluation [3].

One of the notable food additives prior to the emergence of AF-2 was sodium cyclohexylsulfamate (“cyclo”). This substance, which was approved in 1956 as a food additive in Japan, was used worldwide. In 1968, however, the Food and Drug Administration (FDA) of United State announced that some metabolites derived from “cyclo” were responsible for chromosomal damage in experimental animals. In 1969, US government announced that “cyclo” was carcinogenic in experimental animals and prohibited its use [4]. Back again, in 1968, the Ministry of Health and Welfare (MHW) of Japan had organized the Welfare Science Research group and had started investigating the toxicity of cyclohexylamine, one of the metabolites of “cyclo”. Cyclohexylamine was found to induce chromosomal aberrations in cultured human cells, which confirmed the previous report by FDA. In 1969, because of its carcinogenic potential obtained in experimental animals in US, the use and sale of “cyclo” as a food additive was prohibited by the ministerial ordinance of MHW [4].

In 1973, the issue of AF-2 occurred by findings that AF-2 induced mutagenicity and DNA damage in microorganisms and clastogenicity in cultured human lymphocytes and rat bone marrow cells. The use of AF-2 was prohibited officially in 1974 following the demonstration of its carcinogenicity in mice. In response to this, several administrative correspondences were published, including the Interim Standard of Genetic Safety Evaluation of Food Additives issued by the Joint Committee of Toxicity and Food Additives in the Food Sanitation Council. The genotoxicity tests adopted in this standard are as follows [5]:

1. First screening test
  More than two test systems using microorganisms with different characteristics
2. Second screening test
  - Examination of chromosomal aberrations using cultured mammalian cells
  - Examination of sex-linked recessive lethal mutations using *Drosophila* or recessive mutations based on specific-locus method using silkworms
3. In vivo test using mammals
  - Metabolism, and host-mediated assay
  - In vivo chromosomal aberration test
  - Dominant lethal test
  - Specific-locus test using mice
  - Biotransformation

According to the standard, the second screening test should be performed on those substances that show positive results in the first screening test or on those substances that are judged very important (high production volume, wide range of use, and so on), even though negative results were obtained. In the second screening test, chromosomal aberration test using cultured mammalian cells and/or gene mutation tests using insects should be conducted. When substances show positive results in the second screening test or are judged very important, even though negative results were obtained in the second screening test, in vivo tests using mammals should be performed [5].

According to the implementation guidance of this standard, the reverse mutation assay using *S. typhimurium* TA1535, TA1536, TA1537 and TA1538 or *Escherichia coli* WP2 should be conducted in the first screening test, and test organisms such as *E. coli* Sd-4, *Saccharomyces cereviciae* 1765-5A and *Neurospora crassa* ad-3 could be included. In addition, the DNA repair (damage) test using *Bacillus subtilis rec* ^+^/*rec* ^−^ (Rec-assay), *E. coli pol* ^+^/*pol* ^−^, *S. typhimurium hrc* ^+^/*hrc* ^−^ are indicated, and the metabolic activation system using liver homogenates from 2 or 3 mammals such as rats and mice should be adopted in the above tests. For the second screening tests and in vivo tests, some practical recommendations are made in the guidelines, for example, as the use tester strains for the mouse specific-locus test, the PW strain developed by Kiyoshi Tutikawa of the National Institute of Genetics of Japan is described, in addition to the PT strain developed by W. L. Russell and the HT strain developed by M. F. Lyon [5].

In 1973, the MHW founded Carcinogenicity Research Group for technical development of short-term screening tests of chemical carcinogens. In 1975, the research group of Technical Development of Short-Term Screening Tests of Chemical Carcinogens by Utilization of Genotoxicity Tests” (Organizer: T. Kawachi, National Cancer Institute) and the research group of Animal Experiments of Carcinogenicity on Genotoxic Chemicals” (Organizer: S. Odashima, National Institute of Health Sciences) was organized. About 30 substances were evaluated by the Short-Term Screening Tests group for a period of one year, and genotoxicity tests primary used by this group are as follows [6]:

1. Tests using microorganisms
  - DNA repair test using *B. subtilis* (*rec* ^+^/*rec* ^−^) (including metabolic activation) and *E. coli* (WP2, WP100) (liquid and solid method)
  - Reverse mutation assay using *S. typhimurium* (TA100, TA98 and TA1537) (including metabolic activation)
2. Tests using mammalian cells
  - Chromosomal aberration test using Chinese hamster cells (CHL, Don) (including metabolic activation) and human diploid cells (HE2144)
  - Sister chromatid exchange test using Chinese hamster cells (Don) (including metabolic activation) and human diploid cells (HE2144)
  - Micronucleus test using cultured mammalian cells
  - Chromosomal aberration test using rat bone marrow cells
3. Tests using insects
  - Specific-locus test using silkworms
  - Test (M-5 method) using *Drosophila*

According to the guidelines, when test substances showed positive or inconclusive results in the screening group, they were examined by the carcinogenicity test using experimental animals, according to their social demand or production volume. In this way, the usefulness of diverse genotoxicity tests was investigated.

In 1979, the Carcinogenicity Research Group of the MHW tried making a tentative selection from many genotoxicity tests based on the data obtained previously and the trends in overseas as follows [1, 7]:

1. Feasible tests
  - Reverse mutation assay using bacteria
  - DNA repair test using bacteria
  - Chromosomal aberration test using cultured mammalian cells
  - Micronucleus test using mouse bone marrow cells
  - Dominant lethal test using mice or rats.
2. Tests with requirement for further considerations
  - Gene mutation test using bacteria
  - Gene mutation test using insects
  - Gene mutation test using cultured mammalian cells
  - Sister chromatid exchange test using mammalian cells
  - Unscheduled DNA synthesis test using mammalian cells
  - Specific-locus test using mice
  - Reciprocal translocation test using mice or rats
  - Spot test using mice
3. Tests with technical difficulties
  - Chromosomal aberration test using insects
  - Mitotic recombination or gene conversion tests using yeasts
  - Cell transformation test using cultured mammalian cells

## Genotoxicity tests used to establish guidelines

The drug for human use is one of the representatives among various chemical substances. The Thalidomide case in 1961 led to reviewing and strengthening the pharmaceutical regulatory system as well as the guidelines for testing the reproductive toxicity of drugs, which were established in 1965. In 1967, the Basic Policies for Approval to Manufacture Drugs was issued in the form of a notification by the Director General of the Pharmaceutical Affairs Bureau, the MHW [8]. In 1980, the Pharmaceutical Affairs Law was drastically revised, and according to the classification of new drugs, nine different toxicity tests were required, including the genotoxicity test [8]. However, the protocols were not defined, and many requests were issued by Japan and other countries to clarify the guidelines for toxicity testing of drugs.

In 1982, the MHW organized a research group of experts to establish the guidelines for toxicity tests of drugs and undertook a review of the guidelines in Japan, referring to those of other countries [8]. In 1983, draft guidelines for general toxicity tests (acute, subacute, and chronic), carcinogenicity tests, reproductive tests, and genotoxicity tests were published by the MHW. In 1984, the draft guidelines were revised based on the opinions of those concerned with these tests in Japan and in other countries, and the Guidelines for Toxicity Studies of Drugs were established in the form of a notification from the Director General. The tests incorporated a battery of genotoxicity tests as follows: reverse mutation assay using bacteria, chromosomal aberration test using cultured mammalian cells, and micronucleus test using rodents [8]. The number of genotoxicity tests evaluated initially in Japan exceeded 10, of which five were selected as feasible tests that could be performed in Japan. Finally, three tests were chosen based on a discussion of the outcomes of various collaborative studies.

A correlation between chemicals and occupational cancer attracted attention since 1965 and became a public health concern in 1975. Therefore, the Industrial Safety and Health Law was established and enforced by Ministry of Labor (at that time), and safety measures for protection of people from occupational diseases were implemented. According to this law, prior to new chemicals being introduced into the workplace, these compounds should first be examined for adverse effects, and commensurate preventive measures should be performed. However, sufficient effective outcomes were not achieved because a definite regulatory obligation was not offered to companies. Therefore, the Industrial Safety and Health Law was revised and enforced in 1977 and implemented in 1979 [9]. The revised law required companies to examine the adverse effects of new chemicals and report them to the Ministry of Labor.

The law requires that the genotoxicity test or an equivalent or superior test should be performed to evaluate the chemical’s carcinogenicity, instead of the carcinogenicity test using experimental animals. Almost all companies performed the genotoxicity tests, and their outcomes were reported to the Ministry of Labor. The Ministry of Labor obtained opinion from experts regarding the reverse mutation assay as the most appropriate test, because the assay was found to be quick, simple and reproducible. Therefore, in 1979, the Ministry of Labor issued the Standards for Genotoxicity Tests using Microorganisms [9]. According to the knowledge accumulated using this assay and the Guidelines for the Testing of Chemicals and Good Laboratory Practice by OECD, the Ministry of Labor revised the standard according to opinions issued by meetings of experts and released the Standards for Mutagenicity Tests using Microorganism in 1985 [10].

During 1970s, environmental pollution caused by PCB became a serious problem. This issue led to the reconsideration of awareness of chemical safety, and the Act on the Evaluation of Chemical Substances and Regulation of Their Manufacture, was enforced in 1973 as the world’s first and implemented in 1974. Chemicals such as PCB with high bioaccumulation, non-biodegradation, and chronic toxicity should be designated as the Specified Chemical Substance for the implementation of required regulations.

The list of tests indicated at first did not include the genotoxicity test that was added in 1984 without any explanation of test protocols [11]. Moreover, the contamination of groundwater with trichloroethylene occurred in Japan, and this chemical was out of subjects to the regulation of this law, requiring the correspondence for such chemicals. To response to these changing circumstances, the law was drastically revised, which was implemented in 1987 [11]. According to this revision, the determination of the Designated Chemical Substance with low bioaccumulation but non-biodegradation and suspicious chronic toxicity should be performed simultaneously. Furthermore, the genotoxicity test and 28-day repeat-dose toxicity test were introduced for the determination of the Designated Chemical Substances. The reverse mutation assay using bacteria and chromosomal aberration test using cultured mammalian cells were adopted as the genotoxicity test, and these tests together with the 28-day repeat-dose toxicity test using experimental animals were generally referred as the Screening Toxicity Tests of New Chemical Substances.

Consideration of regulations for the substances such as agricultural chemicals, of which categories had been already regulated by other laws, was omitted.

## Conclusion

**Fig. 1:**
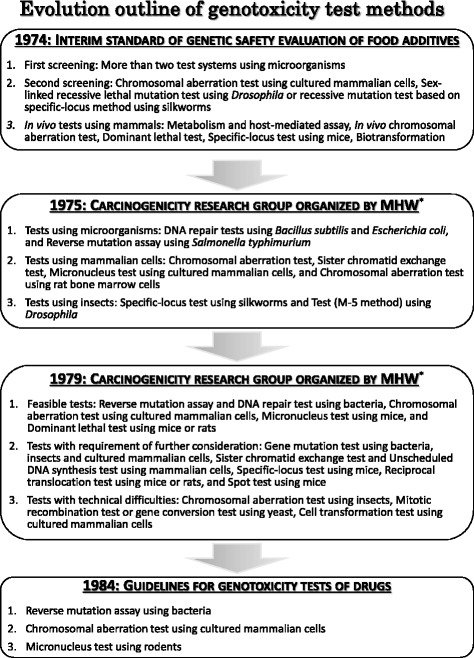
Evolution outline of genotoxicity test methods. *MHW: Ministry of Health and Welfare, which was changed to Ministry of Health, Labour, and Welfare since 2001, based on the reorganization

Outlines of the evolution of genotoxicity test methods are described above and essential items are shown in Fig. 1. In the early stages, various tests were evaluated, and the selection of appropriate ones was difficult considerably. Information on this topic in countries other than Japan is not included here; however, this information has significantly affected the selection of suitable tests in Japan. Accumulation of data is ongoing, and based on such data, revisions of test methodologies are being performed, and new tests are being established. Initially, the genotoxicity test was considered as the screening method for evaluating chemical carcinogens; however, the significance of genotoxicity is the focus of attention, because the mechanisms underlying their toxicological effects are becoming better understood. Moreover, discussions concerning how we can correlate genotoxicity and risk assessment of humans are ongoing. Accordingly, the degree of requirements are higher than before, and it is, therefore, necessary to improve test methodologies, that is, the evolution of genotoxicity test methods will continue.
